# Different Effects of Strong-Bonded Water with Different Degrees of Substitution of Sodium Sulfobutylether-β-cyclodextrin on Encapsulation

**DOI:** 10.3390/pharmaceutics16070919

**Published:** 2024-07-10

**Authors:** Xiaofeng Wang, Jiaqi Huang, Dengchen Yang, Ting Huang, Yang Yang, Jiasheng Tu, Jian Zou, Huimin Sun, Xia Zhao, Rui Yang

**Affiliations:** 1 NMPA Key Laboratory for Quality Research and Evaluation of Pharmaceutical Excipients, National Institutes for Food and Drug Control, Beijing 100050, China; 2 Center for Research Development and Evaluation of Pharmaceutical Excipients and Generic Drugs, Department of Pharmaceutics, School of Pharmacy, China Pharmaceutical University, Nanjing 210009, China; 3 Beijing National Laboratory for Molecular Sciences, CAS Key Laboratory of Organic Solids, Institute of Chemistry, Chinese Academy of Sciences, Beijing 100190, China

**Keywords:** sodium sulfobutylether-β-cyclodextrin, degree of substitution, water, encapsulation

## Abstract

The encapsulation of sodium sulfobutylether-β-cyclodextrin (SBE-β-CD) is influenced not only by the degree of substitution (DS) but also by the presence of strong-bonded water (SBW). Guests compete with SBW for positions within the cavity of SBE-β-CD. However, the correlation between DS and SBW was not clear. This study revealed a positive correlation between DS and SBW utilizing Karl Fischer titration. The mechanism may be attributed to molecular polarizability. To explore the impact of SBW inside SBE-β-CD with different DS on encapsulation, density functional theory was employed. Throughout the release process, an increase in enthalpy is unfavorable, while an increase in entropy favors spontaneous reaction occurrence. For SBE-β-CD (DS = 2, 3), enthalpy increase is the primary factor, leading to the retention of SBW within the cavities and consequently hindering guest entry. In contrast, for SBE-β-CD (DS = 4, 7), the situation differs. For SBE_10_-β-CD, the influence of SBW is minimal. This study aims to elucidate the relationship between DS and SBW, as well as the effect of SBW inside SBE-β-CD with different DS on encapsulation. It is crucial for a comprehensive understanding of the factors affecting the encapsulation of SBE-β-CD, thereby promoting quality control and functional development of SBE-β-CD.

## 1. Introduction

Sulfobutylether-β-cyclodextrin sodium (SBE-β-CD) is an anionic derivative of cyclodextrin (CD) with high water solubility [1,2,3]. It is produced by substituting hydroxyl groups at positions 2, 3, and 6 of β-CD with sulfobutyl groups [4]. The stereostructure of SBE-β-CD resembles a truncated cone, with an internal hydrophobic cavity. The charges of the sulfobutyl groups repel each other, causing them to extend away from the β-CD backbone [5]. Near the β-CD framework, they form a hydrophobic cavity composed of alkyl ether portions. This cavity plays a role in encapsulating hydrophobic or partially hydrophobic guest molecules. The encapsulation of SBE-β-CD with guests primarily relies on non-covalent interactions, including hydrophobic forces [6,7], van der Waals forces [8], and others [9,10]. In the field of pharmaceutics, SBE-β-CD is commonly used as a co-solvent [11,12] and stabilizer [13]. When poorly soluble drugs are encapsulated by SBE-β-CD, their solubility is significantly enhanced. Additionally, unstable guests, upon encapsulation by SBE-β-CD, experience reduced exposure to the external environment to increase stability. Furthermore, SBE-β-CD has additional applications such as adjusting the pK_a_ of drugs [14], enhancing the fluorescence emission of dyes upon encapsulation [15,16,17], modifying sensors to improve sensitivity [18,19], and serving as a chiral selector for separating chiral substances [20,21]. The encapsulation of SBE-β-CD is characterized by reversibility, selectivity, and competitiveness, granting it boundless potential for applications owing to its distinctive attributes.

Currently, commercialized SBE-β-CD include Captisol^®^ and Dexolve^®^, among others. The FDA has approved the use of SBE-β-CD in over 14 drugs. The commercialized products available are mixtures of SBE-β-CDs with varying degrees of substitution (DS). DS indicates the number of sulfobutyl side chains on the β-cyclodextrin backbone (SBE_n_-β-CD represents SBE-β-CD with the DS of n). For commercialized SBE-β-CDs, DS represents the weighted average of each SBE-β-CD. According to US Pharmacopeia (USP) 2023, the DS for SBE-β-CD ranges from 6.2 to 6.9 [22]. Furthermore, it outlines the limit ranges for each component (refer to Table 1), which indicates the distribution of DS. From the distribution of DS, it can be observed that the predominant components in commercially available products are still SBE-β-CD with DS ranging from 4 to 9. From a safety standpoint, DS has a significant impact on hemolytic activity, with the hemolytic order being SBE_7_-β-CD < SBE_4_-β-CD << SBE_1_-β-CD [23]. From a functional standpoint, DS also affects the binding with guests. Traditional studies suggest that this phenomenon is due to the increase in sulfobutyl side chains. On the one hand, the additional side chains extend the hydrophobic cavity. On the other hand, the negatively charged side chains contribute to increased charge interactions. Ashwinkumar et al. conducted experiments and found that the higher the degree of substitution, the higher the solubility and stability constant of danazol [24]. Similarly, Goutam et al. conducted a similar study and attributed the reason to the increase in side chains, gradually strengthening electrostatic interactions [25].

In addition to DS, the presence of strong-bonded water (SBW) also affects the encapsulation of SBE-β-CD with guests [26,27,28]. SBW refers to water molecules within the hydrophobic cavity of SBE-β-CD, which are influenced by the production process of SBE-β-CD. When guests enter CD or CD derivatives to form inclusion complexes, SBW within the hydrophobic cavity is either partially or fully released. The enthalpy and entropy changes of this process influence the formation of subsequent inclusion complexes. Computational methods, such as density functional theory (DFT) [10,29], are commonly used to study this process. The M06-2X density functionals [30], although computationally demanding, offer high accuracy and provide results with practical significance.

**Figure 1:**
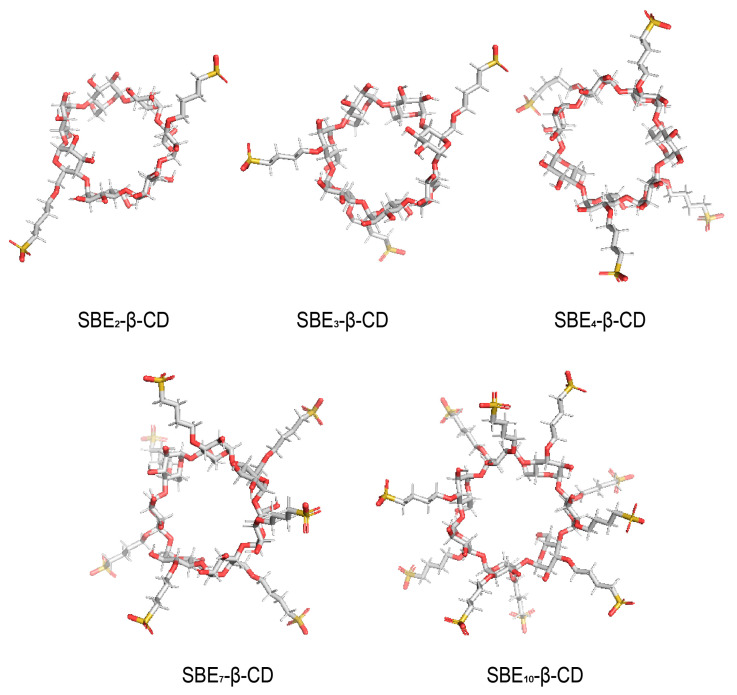
The structure of SBE-β-CD (DS = 2, 3, 4, 7, 10).

Many studies have indicated that both DS and SBW can influence the encapsulation of SBE-β-CD. However, there is a lack of literature reporting on the relationship between these two influencing factors. The consistency of the impact of strong water binding in SBE-β-CD with different DS on encapsulation has been a longstanding gap in the research field. In order to investigate the relationship between DS and SBW, the study quantified the water content of SBE-β-CD (DS ≈ 2, 3, 4, 7, 10), including both total water and SBW through Karl Fischer titration (KFT) [31]. Also, we employed M06-2X density functionals for computational theoretical simulations to clarify the internal connection. Understanding the mechanisms by which SBW in SBE-β-CD (DS = 2, 3, 4, 7, 10) (refer to Figure 1) impacts encapsulation at the molecular level contributes to enhanced quality control and application of SBE-β-CD. Furthermore, this mechanism also holds implications for other macrocyclic molecules with encapsulation capabilities.

## 2. Materials and Methods

### 2.1. Reagents and Materials

SBE-β-CDs (DS ≈ 2, 3, 4, 7, 10) were provided by Zibo Qianhui Biotechnology Company Limited, Zibo, China. Methanol anhydrous (AR, ≥99.7%) and butanol (AR, ≥99.8%) were purchased from Sinopharm Chemical Reagent Company Limited, Shanghai, China. Molecular sieves, 3A (4 mm–6 mm), were achieved from Shanghai Aladdin Biochemical Technology Company Limited, Shanghai, China. CombiTitrant 5 was obtained from Sigma-Aldrich, Darmstadt, Germany.

### 2.2. KFT

Water determination [32] for SBE-β-CDs was realized by the one-component technique KFT. KFT was performed by using a Karl Fischer 901 Titrando apparatus (Metrohm AG, Herisau, Switzerland) and an 803 Ti Stand mixing system (Metrohm AG, Herisau, Switzerland). For acquisition and data handling the Tiamo™, ver. 2.5 software was used. CombiTitrant 5 was used for KFT. Additional solvents were methanol anhydrous and butanol (dried by molecular sieves, 3 A). The sample amount was 0.10–0.30 g for SBE-β-CDs. The method parameters were I(pol) of 50 lA, endpoint and dynamics at 250 mV, and drift was used as the stop criterion, with a stop drift of 20 μL/min. For titration parameters, the maximum extraction time was 20 s, time interval measuring point of 2 s. The temperature was set up to 25 °C. The conditioning was set up for a spot volume of 20 mL, with a delay after ‘‘conditioning ok’’ of 8 s.

### 2.3. Computational Details

Density functional theory research was conducted on the structures of SBE-β-CDs (DS = 2, 3, 4, 7.10) and a fixed number of water molecules. All calculations were conducted with the Gaussian 16 software package [33]. Geometry optimizations were carried out in the gas phase using the M06-2X [34] functional with the 2-ζ basis set 6-31G(d,p) [35,36,37,38]. Frequency calculations were performed at the same level of theory to identify all the stationary points as minima (zero imaginary frequencies). The relative enthalpy energies for all structures were refined by performing single-point energy calculations with the larger 6-311++G(d,p) for all atoms [39]. All structures are calculated in the gas phase. Next, different SBE-β-CDs were kinetically optimized by adding 50 times water to GFN2-XTB in the ab initio molecular dynamics method (AIMD) [40]. The final known position of the water molecule was determined by referring to the study of Stiliyana Pereva et al. [30]. Finally, the same method is used to calculate the structural optimization and single-point energy for the water-containing structure.

## 3. Results

### 3.1. The Positive Correlation between the Content of SBW and DS

**Figure 2:**
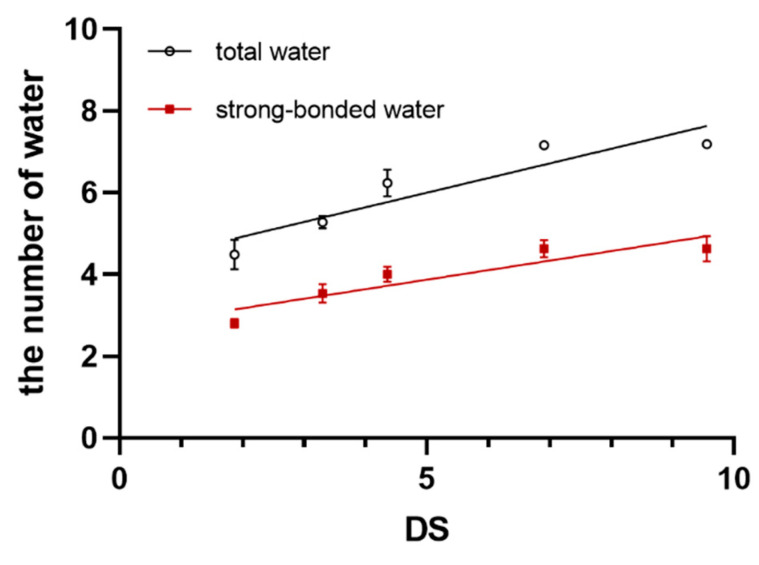
The correlation plot illustrating the relationship between the DS of SBE-β-CD and both the total number of water and the quantity of SBW.

KFT was used to determine the water content and SBW content of SBE-β-CDs (DS ≈ 2, 3, 4, 7, 10). Based on the average molecular weight, the average number of water molecules and SBW molecules per SBE-β-CD molecule can be calculated (refer to Table 2 and Figure 2). For SBE-β-CD with DS around 2, 3, 4, 7, and 10, the total number of water molecules is approximately 4.49, 5.38, 6.24, 7.16, and 7.19, respectively. The corresponding numbers of SBW molecules for SBE-β-CD with DS approximately 2, 3, 4, 7, and 10 are 2.80, 3.54, 4.00, 4.63, and 4.63, respectively. Analysis of variance (ANOVA) on this result showed significant differences (*p* < 0.05) in the total number of water molecules and SBW molecules among SBE-β-CDs with different DS. This indicates that SBE-β-CDs with different DS exhibit variability in the number of total water molecules and SBW molecules.

Additionally, Pearson correlation analysis was conducted on the results. The Pearson correlation coefficient between the number of total water molecules and the degree of substitution was 0.911 (*p* < 0.01), indicating a significant positive correlation between the total number of water molecules and DS. The Pearson correlation coefficient between the number of SBW molecules and DS was 0.885 (*p* < 0.01), suggesting a significant positive correlation between the number of SBW molecules and DS. This finding indicates that under the same production process, the DS of SBE-β-CD is positively correlated with both the total water content and the content of SBW.

Under the same manufacturing process, there exists a positive correlation between the DS of SBE-β-CD and its total water content. This finding implies that manufacturers could utilize water content as one of the reference criteria for preliminary assessment of batch-to-batch consistency in products. Compared to capillary electrophoresis for determining DS of SBE-β-CD, water content measurement offers a simpler and more efficient alternative to enhance the efficiency of quality evaluation.

Traditional theories suggest that both DS and SBW affect the functionality of SBE-β-CD independently, but the underlying connection between these two parameters remains unclear. This study fills this gap by providing insights into their relationship. DS and SBW can directly influence the encapsulation ability of SBE-β-CD. Moreover, DS can also impact the encapsulation ability of SBE-β-CD by affecting the quantity of SBW.

### 3.2. The Mechanism of DS Affecting the Content of SBW

**Figure 3:**
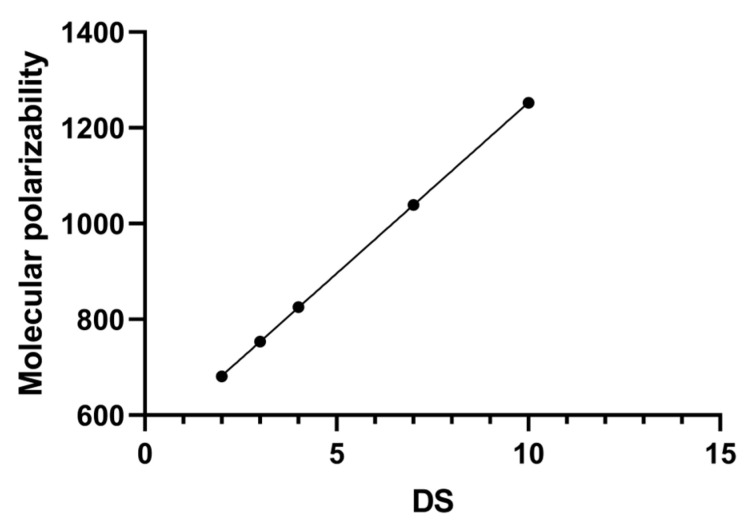
The relationship between DS of SBE-β-CD and molecular polarizability.

To explore the mechanism behind the increase in SBW with DS, computational simulations were conducted. Models of SBE-β-CDs with different DS were established for structural optimization and single-point energy calculations. The exact polarizability of these models was obtained from the output results. Simulation results indicate a linear increase in molecular polarizability with increasing DS, as depicted in Figure 3. These findings confirm that the increase in DS leads to an increase in molecular polarizability. Studies suggest that the augmentation in molecular polarizability facilitates hydrogen bond formation and enhances hygroscopicity [24]. Greater polarizability enables easier deformation of electron clouds, facilitating hydrogen bond formation. Hence, the formation of SBW becomes more facile. Thus, the mechanism through which DS influences the content of SBW may be attributed to the variation in polarizability with DS.

### 3.3. The Influence of SBW on Encapsulation

Before the guest enters the hydrophobic cavity, SBW is released either partially or completely, impacting the encapsulation of SBE-β-CD. Considering the competition between the guest molecule and SBW for SBE-β-CD cavity occupancy, this study aims to further explore the influence of DS on the release of SBW in SBE-β-CD. On the one hand, different quantities of SBW are simulated to be released from SBE-β-CD with a specific DS to investigate the effect of SBW quantity on its release. On the other hand, the release of SBW within SBE-β-CD of varying DS is simulated to demonstrate how DS affects the release of SBW, thereby influencing the encapsulation ability of SBE-β-CD. The simulation yields changes in enthalpy (ΔH) and entropy (ΔS) during the release process. Utilizing the Gibbs free energy formula (ΔG = ΔH − TΔS), the Gibbs free energy (ΔG) of the process at room temperature (T = 298.15 K) can be calculated, allowing assessment of the process.

#### 3.3.1. The Impact of the Quantity of SBW on the Encapsulation of SBE_7_-β-CD

**Figure 4:**
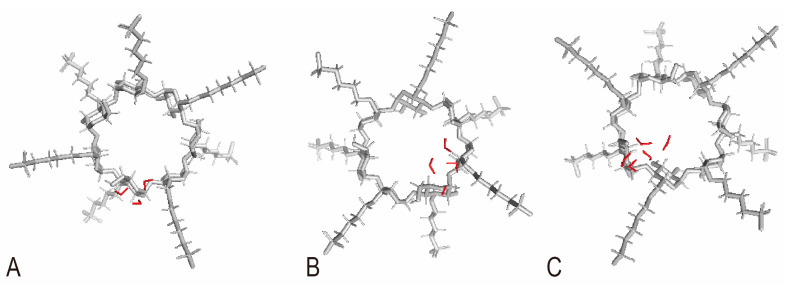
The positions of SBW within SBE_7_-β-CD: (**A**) SBE_7_-β-CD + 3H_2_O; (**B**) SBE_7_-β-CD + 4H_2_O; (**C**) SBE_7_-β-CD + 5H_2_O.

To investigate the impact of the quantity of SBW on the encapsulation ability of SBE-β-CD, simulations were conducted to simulate the release process of 3, 4, and 5 SBW molecules within SBE_7_-β-CD (refer to Table 3 and Figure 4). SBE_7_-β-CD was chosen because it represents the approximate DS of commercially available SBE-β-CD. The simulation was conducted with 3, 4, and 5 SBW molecules because the range of SBW quantities determined by KFT falls within this range, from 3 to 5 molecules.

The ΔH and ΔS values for the release of SBW within SBE_7_-β-CD were both positive, indicating energy absorption during the separation process. An increase in enthalpy is unfavorable for the reaction, while an increase in entropy is favorable. When ΔH and ΔS act in opposite directions, the spontaneity of the reaction depends on the dominant factor. At 298.15 K, the calculated ΔG for the release processes of 3, 4, and 5 water molecules were all negative, indicating spontaneous reactions. This also suggests that for SBE_7_-β-CD, the release of SBW is dominated by entropy increase. When there are 3, 4, or 5 water molecules in the hydrophobic cavity of SBE_7_-β-CD, the water molecules tend to spontaneously release from the hydrophobic cavity. After some or all of the water molecules are released, guests can enter the SBE_7_-β-CD cavity. Therefore, when the quantity of SBW is between 3 and 5, the dominant factor is ΔS. Currently, no evidence suggests that changes in the quantity of SBW alter the propensity for spontaneous release within SBE_7_-β-CD.

#### 3.3.2. The Effect of SBW in SBE-β-CD with Various DS on Encapsulation

**Figure 5:**
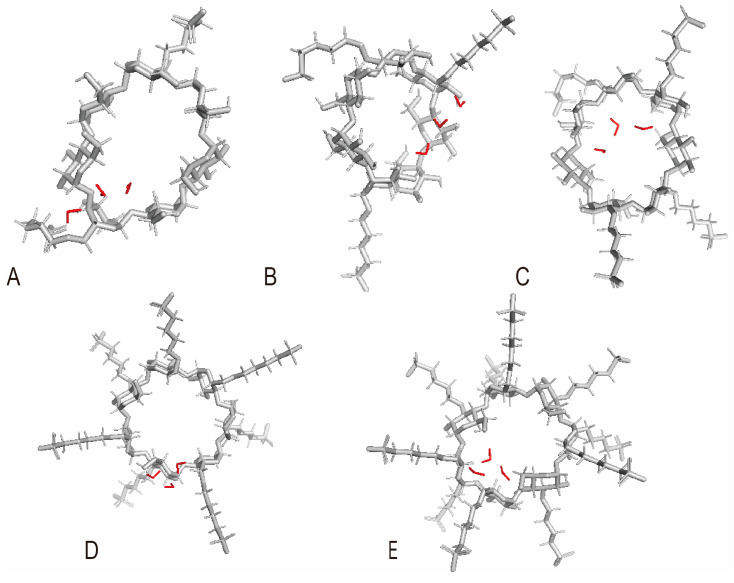
The positions of SBW molecules within SBE-β-CD with varying DS: (**A**) SBE_2_-β-CD + 3H_2_O; (**B**) SBE_3_-β-CD + 3H_2_O; (**C**) SBE_4_-β-CD + 3H_2_O; (**D**) SBE_7_-β-CD + 3H_2_O; (**E**) SBE_10_-β-CD + 3H_2_O.

To investigate the effect of DS on the release of SBW from the SBE-β-CD, simulations were conducted for SBE-β-CD with different DS (DS = 2, 3, 4, 7, 10) separating from an equal number of water molecules (refer to Table 4 and Figure 5). The number of simulated water molecules was set to 3, as the minimum quantity of SBW determined by KFT is 3. The study revealed that the impact of SBW varies for SBE-β-CD with different DS. For SBE-β-CD (DS = 2, 3, 4, 7, 10), both the enthalpy change (ΔH) and the entropy change (ΔS) during the release process of SBW from the internal cavity were positive. This indicates energy absorption during the release process, similar to the energy changes observed in the release process of SBW within SBE_7_-β-CD. An increase in enthalpy hinders the progress of the reaction, while an increase in entropy favors it. When these two effects oppose each other, whether the reaction proceeds spontaneously depends on the dominant factor.

For SBE_2_-β-CD and SBE_3_-β-CD, ΔG during the release process of SBW from the interior is greater than 0, indicating that the process is not spontaneous. This suggests that the process is dominated by enthalpy increase. SBW tends to remain inside the cavity, occupying part of the hydrophobic cavity space, making it more difficult for guests to enter the SBE-β-CD. For SBE_4_-β-CD and SBE_7_-β-CD, ΔG is less than 0, indicating that the process is spontaneous. This suggests that the process is dominated by entropy increase. SBW tends to come out of the SBE-β-CD cavity. When water partially or completely exits, guests can enter the SBE-β-CD cavity, facilitating the inclusion process. As for SBE_10_-β-CD, compared to SBE-β-CD with other DS, ΔG of SBW binding to SBE-β-CD is positive but very close to 0, indicating that the binding and release of SBW in the hydrophobic cavity are close to equilibrium. Therefore, the influence of SBW on guest entry into the hydrophobic cavity of SBE_10_-β-CD is small, meaning its impact on the inclusion process of SBE_10_-β-CD is minimal.

In essence, for SBE-β-CD with lower DS, the predominant factor governing the release of SBW from the hydrophobic cavity is an increase in enthalpy, rendering it less prone to spontaneous occurrence and unfavorably impacting the encapsulation process. Conversely, for SBE-β-CD with moderate DS, the release of SBW is influenced by an increase in entropy, facilitating its spontaneous release and enhancing encapsulation. For SBE-β-CD with higher DS, the effect of SBW on encapsulation ability is minimal. This finding provides a more theoretical foundation for establishing the limit range of each SBE-β-CD from a functional standpoint. Understanding the influence of internal SBW on encapsulation for SBE-β-CD with varying DS is imperative for advancing its quality control and application at a deeper level. Furthermore, it presents a novel approach for adjusting the encapsulation capability of other large-ring molecules with similar characteristics.

## 4. Discussion

The research found that the DS of SBE-β-CD correlated positively with both the number of total water molecules and the amount of SBW under the same production process. The mechanism underlying the aforementioned positive correlation may be related to the increasing molecular polarizability with DS. In the process of releasing SBW within the cavities of SBE-β-CD, an increase in enthalpy impedes the reaction, whereas an increase in entropy promotes the reaction to occur spontaneously. The primary factor governing the release process of SBW differs among SBE-β-CDs with varying DS. For SBE_2_-β-CD and SBE_3_-β-CD, enthalpy increase is the primary factor, leading to the retention of SBW within the cavities and consequently hindering guest entry. In contrast, for SBE_4_-β-CD and SBE_7_-β-CD, the situation differs. For SBE_10_-β-CD, the influence of SBW is minimal.

## 5. Conclusions

Both DS and the presence of SBW have an impact on the encapsulation of SBE-β-CD. According to the research, DS and the presence of SBW within the cavity are not independent factors influencing the encapsulation of SBE-β-CD. Not only is there a positive correlation between DS and the quantity of SBW, possibly due to molecular polarizability, but also the dominant factor in the process of SBW release is closely related to DS. During the process of SBW release, an increase in enthalpy is unfavorable for the reaction, while an increase in entropy favors the spontaneous progression of the reaction. For SBE-β-CD with lower DS, the presence of SBW impedes the entry of guest molecules into the SBE-β-CD cavity. For SBE-β-CD with moderate DS, the presence of SBW facilitates the entry of guest molecules into the SBE-β-CD cavity. For SBE-β-CD with higher DS, the influence of SBW on the entry of guest molecules into the SBE-β-CD cavity is minimal.

SBE-β-CD is widely utilized in pharmaceutics, pharmaceutical analysis, and biomedical engineering due to its excellent encapsulation properties. Understanding the factors influencing encapsulation is beneficial for the in-depth application and quality control of SBE-β-CD.

According to the USP, the allowable content of water in SBE-β-CD should not be greater than 10%. From the perspective of the encapsulation of SBE-β-CD, the relationship between total water content and SBW quantity is a topic worthy of exploration. SBE-β-CD exhibits strong hygroscopicity, and it is worth investigating whether the quantity of SBW in SBE-β-CD changes after absorbing moisture, affecting its encapsulation. This is a direction we will continue to explore in our future research endeavors.

## Figures and Tables

**Table 1:** The limit range of each SBE-β-CD.

DS	Limit Range (%)
1	0–0.3
2	0–0.9
3	0.5–5.0
4	2.0–10.0
5	10.0–20.0
6	15.0–25.0
7	20.0–30.0
8	10.0–25.0
9	2.0–12.0
10	0–4.0

**Table 2:** The number of the total water and SBW in SBE-β-CD with different DS.

	DS (Average ± Standard Deviation) (*n* = 3)					*F*	*p*	Pearson Correlation Coefficient
	1.87	3.30	4.36	6.91	9.56			
Total water	4.49 ± 0.36	5.28 ± 0.15	6.24 ± 0.33	7.16 ± 0.04	7.19 ± 0.04	78.886	0.000 **	0.911 **
SBW	2.80 ± 0.11	3.54 ± 0.23	4.00 ± 0.19	4.63 ± 0.21	4.63 ± 0.31	37.030	0.000 **	0.885 **

** *p* < 0.01.

**Table 3:** The energy changes associated with the release of different quantities of SBW within SBE_7_-β-CD.

The Number of SBW	3	4	5
ΔH (cal/mol)	27,945.3922	38,470.6708	47,125.2312
ΔS (cal/mol)	98.7140	137.1910	161.9910
ΔG (cal/mol)	−1486	−2433	−1172

**Table 4:** The energy change associated with the release of three water molecules from SBE-β-CD with different DS.

DS	2	3	4	7	10
ΔH (cal/mol)	58,464.1512	49,554.6063	31,432.1753	27,945.3922	31,805.9224
ΔS (cal/mol)	122.3380	115.0210	106.6270	98.7140	106.6240
ΔG (cal/mol)	21,989	15,261	−359	−1486	16

## Data Availability

The data used to support the findings of this study are available from the corresponding author upon request.
